# Rethinking Social Participation Services and Interventions: Innovative Approaches for Diverse Aging Realities

**DOI:** 10.1093/geroni/igaf122.772

**Published:** 2025-12-31

**Authors:** Mélanie Levasseur, Dolores Majon-Valpuesta

## Abstract

As a key determinant of health, particularly in later life when the risk of social isolation and exclusion increases, social participation and connection requires effective services and interventions addressing the diverse needs, life conditions and trajectories of aging populations. This symposium aims to present innovative services and interventions designed to meet older adults’ specific participation and connection needs, improve access to resources, and foster their active engagement in the community according to diverse aging realities. Drawing on theorical and empirical evidence, it will provide a forum for broader reflection and discussion on how to strengthen the participation and connection opportunities for older adults by proposing interventions that range from taking advantage of technological advances to favoring self-questioning of services. Levasseur and colleagues explored experiences of older adults interacting with one social robot having the ability to converse and promote social participation. Majón-Valpuesta and colleagues considered the strategies to update services aimed to promote social participation considering the baby boomer generation’s needs. Lévesque and colleagues examined the compatibility of social prescribing by preventive occupational therapy intervention to expand older adults’ access to meaningful and health-promoting activities. Lord and colleagues, identified factors associated with and counteracting epistemic injustices to innovate in health services, better supporting social participation and connection. Together, these presentations foster interdisciplinary dialogue between earlier and mid-career as well as senior researchers, bringing diverse perspectives to help shape policy, practice, and advocacy that strengthen innovation in social participation and connection, and better support the health and well-being of aging populations.

